# Determining the Appropriate Treatment for T-Cell Acute Lymphoblastic Leukemia With *SET-CAN/NUP214* Fusion: Perspectives From a Case Report and Literature Review

**DOI:** 10.3389/fonc.2021.651494

**Published:** 2021-03-26

**Authors:** Na Lin, Zhenghua Liu, Yan Li, Xiaojing Yan, Lei Wang

**Affiliations:** Department of Hematology, The First Affiliated Hospital of China Medical University, Shenyang, China

**Keywords:** *SET-CAN/NUP214* fusion, T-cell acute lymphoblastic leukemia, treatment, allogenic hematopoietic stem cell transplantation, prognosis

## Abstract

*SET-CAN/NUP214* fusion is a recurrent event most commonly seen in T-cell acute lymphoblastic leukemia (T-ALL). It is related to resistance to glucocorticoids and chemotherapy; however, the reported prognosis of T-ALL with *SET-CAN/NUP214* fusion is diverse, and the optimal treatment option remains undetermined. Here, we present the treatment process of an illuminating case of T-ALL with *SET-CAN/NUP214* fusion. The patient showed early resistance to routine VICLP chemotherapy (at 15^th^ day, 79.2% blasts), but the leukemia burden was significantly reduced after 28-day induction chemotherapy (18.85% blasts), even though she still didn’t achieve complete remission (CR) after a second course of high-dose methotrexate (3 g/m2) and pegaspargase. Ex vivo drug sensitivity screening using a panel of 165 kinds of cytotoxic drugs, targeted therapy drugs, combination chemotherapy drugs, etc., was conducted on the refractory leukemia cells, which showed extensive resistance to various regimens. Surprisingly, AML-like scheme DAE scheme (daunorubicin + cytarabine + etoposide) and carfilzomib showed the highest ex vivo inhibition rate. The patient received DAE regimen chemotherapy, and finally achieved complete remission and received allogenic hematopoietic stem cell transplantation (allo-HSCT). According to our own findings and a literature survey, we found that T-ALL patients with *SET-CAN/NUP214* fusion usually shows early resistance to chemotherapy, but they have a delayed response, and the CR rate is not compromised; thus, a chemotherapy regimen featuring a 28-day long course, such as that used in GRAALL 2003 or 2005, is recommended for induction therapy. For refractory patients, AML-like therapy such as DAE or CLAG in combination with asparaginase may be beneficial. In addition, carfilzomib may be a useful therapeutic drug and is worthy of further study. Allo-HSCT improves prognosis and we recommend HSCT if possible. Additional chromosomal or molecular events may affect the prognosis, and further investigation is needed. We believe that through proper treatment, the prognosis of patients with *SET-CAN/NUP214* fusion can be greatly improved, at least not worse than that of other T-ALL patients.

## Introduction

Recurrent genetic abnormalities always provide important information about pathological mechanisms, prognosis and even treatment selections in acute leukemias, such as the *PML/RAR* and *AML/ETO* rearrangements in acute myeloid leukemia (AML). *SET-CAN/NUP214* fusion is formed by cryptic t(9;9)(q34;q34) or del(9)(q34.11q34.13) (1). It is a recurrent event initially detected in a patient with acute undifferentiated leukemia (AUL) (2). Later, it may also be detected in patients with AML (3), B-ALL (4) or myeloid sarcoma (1), but the highest frequency is found in T-ALL (1). Knowledge about T-ALL with *SET-CAN/NUP214* is limited. It is generally believed to be related to resistance to glucocorticoids and chemotherapy and to poor prognosis (4–7). However, one study including 8 cases with *SET-CAN/NUP214* fusion reported a better prognosis (3y OS 87.5%) than that of other T-ALL patients (8). Another study described 11 *SET-CAN/NUP214* -positive T-ALL patients and reported similar prognoses for T-ALL patients without this fusion (73% vs 68%) (9). These findings suggest that the prognosis of T-ALL with *SET-CAN/NUP214* fusion may not be as poor as generally considered and can be greatly improved with proper treatment; accordingly, it is urgent to determine the optimal treatment measures. Here, we present the treatment process of an illuminating case of T-ALL with *SET-CAN/NUP214* fusion. According to our own findings and a literature survey, we summarize the clinical features of the disease and put forward our treatment suggestions. We believe that through proper treatment, prognosis of patients with *SET-CAN/NUP214* fusion can be greatly improved or at least not be worse than that of other T-ALL patients.

## Case Presentation

**Figure 1:**
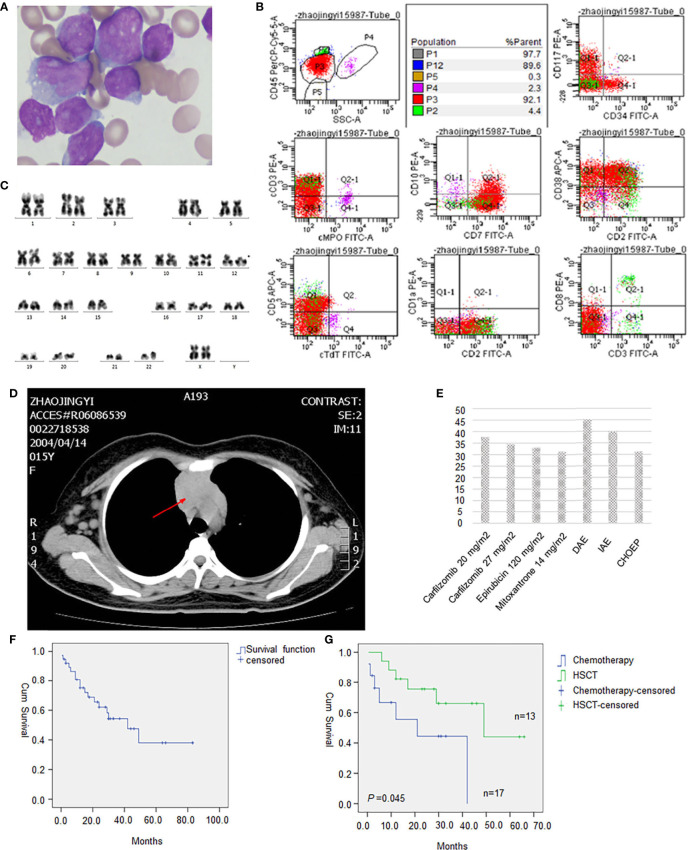
**(A)** Morphology of leukemic cells at diagnosis(original magnification, 1000). **(B)** Flow cytometry result. **(C)** Karyotype analysis showed 46, XX, del(12)(p11)[19]/46, XX[1]. **(D)** Drug sensitivity screening test *in vitro* with a panel of 165 kinds of cytotoxic drugs, molecular targeted therapy drugs, and combination chemotherapy regimens. Relative inhibition rates higher than 30% were listed. **(E)** Chest CT showed an enlarged thymus. **(F)** Survival analysis of all 38 evaluable T-ALL patients with *SET-CAN/NUP214* fusion. **(G)** HSCT significantly improved overall survival of T-ALL patients with *SET-CAN/NUP214* patient compared with chemotherapy.

A 15-year-old girl was admitted to our hospital on October 22, 2019, due to persistent lymph node enlargement for three months. She had no family genetic history. The physical examination showed multiple enlarged lymph nodes in both the neck and groin, which were firm and showed moderate activity with the largest one about 4 cm in diameter. The blood tests showed a white blood cell (WBC) count of 23.5×109/L, a hemoglobin (HGB) level of 106 g/L and a platelet count of 131×109/L. Thirteen percent blasts were found in the peripheral blood. Bone marrow aspiration was performed and revealed hypercellularity with predominant blasts in accordance with L2-type ALL (**Figure 1A**). Flow cytometry (**Figure 1B**) showed that the blasts (P3 group, 92.1%) were mainly positive for CD7, CD38 and CD2; partially positive for CD117, cCD3, CD99, and CD5; weakly positive for CD34 and CD10; and negative for CD33, CD19, CD56, cMPO, cCD79a, CD1a, CD25, HLA-DR, CD15, CD13, cTdT, CD123, CD3, CD8, and CD4, indicating a diagnosis of early T-cell precursor (ETP)-ALL. Karyotyping analysis of the peripheral blood illustrated that the patient had a 46, XX, del(12)(p11)[19]/46,XX[1] karyotype[20] (**Figure 1C**). Reverse transcriptase (RT)-PCR covering 56 commonly detected fusion genes in leukemia (listed in **Supplementary Table 1**) performed on the bone marrow sample detected *SET-CAN/NUP214* gene fusion. Next-generation sequencing (NGS) was performed on 120 commonly mutated genes in ALL (listed in **Supplementary Table 2**), and we identified two *NOTCH1* (NM_017617) mutations: exon 27: c. T5033C (p. L1678P) (4.51%) and exon 26: c.4732_4734del (p.1578_1578del) (19.07%); one *JAK1* (NM_001321852) mutation (1.76%): exon 15: c. G2108T (p. S703I); one *JAK3* (NM_000215) mutation (46.88%): exon 19: c. T2570A (p. L857Q); one *NRAS* (NM_002524) mutation (45.05%): exon 3: c. C176A (p. A59D); and one *DNM2* (NM_001005360) mutation (3.38%): exon 15: c.1620_1621insTACTGGT (p.E540fs). Chest CT showed thymus enlargement (**Figure 1D**), which is a common phenomenon in T-ALL suggesting a possible involvement by leukemia.

The patient received VICLP scheme chemotherapy. On the 14th day, there were still 79.2% blasts in the bone marrow, suggesting resistance to routine chemotherapy. However, after the 28-day full course of chemotherapy, bone marrow blasts were reduced to 18.85% by flowcytometry. A second course of high-dose methotrexate (3 g/m2) and pegaspargase was given, but the patient still failed to achieve CR. Refractory bone marrow leukemia cells were cultured for 72 hours ex vivo, and a panel of 165 kinds of cytotoxic drugs, targeted therapy drugs, combination chemotherapy drugs, etc. (listed in **Supplementary Table 3**), was used for sensitivity screening. This service was provided by Hefei PreceDo Pharmaceuticals Co., Ltd. The results showed that the leukemia cells were extensively resistant to most of the drugs and combinations (**Supplementary Table 3**). Those treatments that achieved an inhibition rate higher than 30% are listed in **Figure 1E**. The patient received the DAE scheme (daunorubicin 40mg/m2 day 1st-3rd + cytarabine 100mg/m2 day 1st-7th + etoposide 100mg/m2 day 1st -5th), which showed highest ex vivo inhibition rate of 45.19%, and ultimately achieved complete remission with only 0.64% residual leukemia cells detected by flowcytometry. Then a sequential consolidation chemotherapies of EAD scheme (etoposide 100mg/m2 day 1st -3rd + cytarabine 3g Q12H day 1st -3rd +dexamethasone 30mg day 1st -3rd), and Hypr-CVAD A/B scheme were given to gain a deeper remission. However there were still a residual of 0.08% of SET-CAN/NUP214 fusion by RT-PCR before allo-HSCT, which turned negative only after allo-HSCT, which was conducted seven months after diagnosis. After HSCT, the patient was followed up regularly every 1-2 months. In the latest follow-up in February 2021, the patient was still in leukemia free state, with a negative SET-CAN/NUP214 fusion by RT-PCR.

## Discussion

T-cell acute lymphoblastic leukemia (T-ALL) with *SET-CAN/NUP214* fusion was relatively rare; it is most often reported in case reports or small-size case series. By reviewing the literature, we found 84 T-ALL patients with *SET-CAN/NUP214* (including this case) (1, 6–25); the clinical characteristics are provided in **Table 1**. Sixty-six patients with detailed information are listed in **Supplementary Table 4**. The proportion of SET-CAN fusion in T-ALL is reported variable. We summarized all reported data and found that the incidence in children is lower than that in adults (2.77% vs 6.30%, *P*=0.005). Most patients are male (76.19%). 16.67% patients have a WBC count higher than 100×109/L.

**Table 1:** Clinical characteristics of T-ALL with *SET-CAN/NUP214* fusion.

Case characteristics	Case number(positive/total)	Percentage
Proportion (SET-CAN/T-ALL)		
Children	14/505	2.77%
Adults	41/651	6.30%
Gender		
Male	48/63	76.19%
Female	15/63	23.81%
Age		
<18	16/64	25%
≥18	48/64	75%
WBC		
<100*10^9/L	40/48	83.33%
≥100*10^9/L	8/48	16.67%
Complete remission	26/36	72.22%
Immunophenotype		
CD33 (positive/all)	23/34	67.6%
CD34 (positive/all)	27/34	79.4%
Karyotype and FISH		
Abnormal 12p	14/52	26.92%
Complex karyotype	16/49	32.65%
Concurrently	12/49	24.49%

Here, we present a T-ALL patient with *SET-CAN/NUP214* fusion with del(12)(p11) and multiple gene mutations. In the early stage, on the 14^th^ day of induction chemotherapy, 79.2% of blasts remained in the bone marrow, which indicated resistance to chemotherapy. However, leukemia cells dropped to 18.85% after the entire course of chemotherapy. This result was to a certain extent similar to the results of Ben Abdelali, R. et al. (9), who also found 91% resistance to corticosteroids and 100% resistance to chemotherapy at 7^th^ day but a high complete remission rate of 90.9% (10/11) after an intact induction scheme of GRAALL 2003 or 2005. As shown in **Table 1** and **Supplementary Table 4**, of the 36 assessable patients, the complete remission rate was 72.22% (26/36), similar to that of all T-ALL patients (73%) (26). All of the above infers that *SET-CAN/NUP214* positive leukemia cells show a good response even though there is a delayed response to chemotherapy, so an induction chemotherapy scheme featuring a 28-day long course, such as that used in GRAALL 2003 and 2005, is recommended.

The best treatment options for refractory patients with *SET-CAN/NUP214* fusion are undetermined. Yang et al. (7) suggested that CLAG chemotherapy in combination with asparaginase might be a potential treatment option for these patients. In this study, our drug sensitivity screening and clinical experience also showed potential benefits from DAE, which is mostly used for AML (27). These data suggested that AML-like therapy may benefit refractory patients with *SET-CAN/NUP214* fusion. This may be in part due to the frequent expression of myeloid and stem markers such as CD33 and CD34. As shown in **Table 1**, of the 34 evaluable patients, CD33 and CD34 were expressed in 67.6% (23/34) and 79.4% (27/34), respectively. Notably, carfilzomib monotherapy had an inhibition rate of 37.57%. As a second-generation, irreversible, selective proteasome inhibitor, carfilzomib has been shown in previous studies to have an antileukemic role in T-cell leukemia cell lines (28, 29). Unfortunately, carfilzomib is still clinically unavailable in China, and thus, our patient did not receive carfilzomib therapy.

We further analyzed the survival data in all 38 evaluable patients, and as shown in **Figure 1F**, the median survival was 42 months. Allo-hematopoietic stem cell transplantation (allo-HSCT) significantly improved prognosis (*P*=0.045), with median survival times of 21 months and 49 months in the chemotherapy group (N=13) and HSCT group (N=17), respectively (**Figure 1G**). In Huang, Z. F.’s research, the median survival time was 24 months and 30 months in the chemotherapy group and allo-HSCT group, respectively (26). Therefore, patients with *SET-CAN/NUP214* fusion may benefit more from allo-HSCT than other patient; if possible, we recommend allo-HSCT for consolidation therapy.

It is noteworthy that almost all patients either had additional cytogenetic or molecular genetic changes. Therefore, regardless of the treatment method used, the diverse prognoses of patients are probably a result of the different concomitant cytogenetic and molecular genetic changes present across the disease. In this patient, del(12)(p11) was detected. An abnormal short arm of chromosome 12 is common in 26.92% (14/52) of patients, although mostly as part of the complex karyotype (12/14). *ETV6* located at 12p13 has been reported to be involved in various leukemias, including ETP-ALL (30), and may contribute to leukemogenesis. We tried to compare the prognosis of patients with or without del(12), but no differences were found (*P*>0.05, data not shown). Knowledge about the molecular characteristics of patients with *SET-CAN/NUP214* fusion is scarce. Five studies selectively assessed mutations of *NOTCH1*, *PHF6*, *FBW7*, *WT1*, *NRAS*, *KRAS*, *IKZF*, *JAK1* or *JAK3* by PCR. Only one study assessed genomic mutations by RNA-seq, and two studies (including this case) assessed common recurrent mutations by next-generation sequencing. As shown in **Table 2**, *NOTCH1* mutation is the most frequent mutation occurs in 74.2% patients, which plays important roles in promoting develop and progression of T-ALL. Other common mutations include *PHF6*, *KRAS* and *JAK3*, which all occur in more than 30% patients. In this patient, mutations in *NOTCH1*, *JAK1*, *JAK3*, *NRAS* and *DNM2* were detected, all of which were recurrent mutations in T-ALL with *SET-CAN/NUP214* fusion, besides they have also been repeatedly described in ETP-ALL which is associated with poor prognosis [15]. However, due to the limited number of cases, we failed to find any prognostic value of genetic mutations. More cases need to be accumulated and evaluated.

**Table 2:** Molecular sequencing data of T-ALL patients with *SET-CAN/NUP214* fusion.

Gene	Mutation/Total tested	Positive percentage
*NOTCH1*	23/31	74.2%
*PHF6*	11/21	52.38%
*KRAS*	6/14	42.86%
*JAK3*	4/12	33.33%
*CCND3*	3/12	25%
*JAK1*	3/15	20%
*STAT5B*	2/10	20%
*DNM2*	2/10	20%
*EED*	2/10	20%
*SUZ12*	2/10	20%
*CHD4*	2/10	20%
*MINK1*	2/10	20%
*KMT2D*	2/10	20%
*EZH2*	2/12	16.67%
*NRAS*	2/12	16.67%
*FBW7*	3/21	14.29%

In summary, SET-CAN/NUP214 fusion is a recurrent event commonly seen in male adult T-ALL patients. Although the patients shows early resistance to chemotherapy, they have a delayed response, but the CR rate is not compromised; thus, a chemotherapy regimen featuring a 28-day long course, such as that used in GRAALL 2003 or 2005, is recommended for induction therapy. For refractory patients, AML-like therapy such as DAE or CLAG in combination with asparaginase may be beneficial. In addition, carfilzomib may be a useful therapeutic drug and is worthy of further study. Allo-HSCT improves prognosis and we recommend HSCT if possible. Additional chromosomal or molecular events may affect the prognosis, and further investigation is needed. We believe that through proper treatment, the prognosis of patients with SET-CAN/NUP214 fusion can be greatly improved, at least not worse than that of other T-ALLL patients.

## Data Availability Statement

The original contributions presented in the study are included in the article/**Supplementary Material**. Further inquiries can be directed to the corresponding author.

## Ethics Statement

Ethical review and approval was not required for the study on human participants in accordance with the local legislation and institutional requirements. Written informed consent to participate in this study was provided by the participants’ legal guardian/next of kin. Written informed consent was obtained from the minor(s)’ legal guardian/next of kin for the publication of any potentially identifiable images or data included in this article.

## Author Contributions

NL and YL guided the treatment of this case. NL drafted the manuscript. ZL and LW reviewed all related literature. XY and LW interpreted data and critically revised the manuscript. All authors contributed to the article and approved the submitted version.

## Funding

This study was funded by the National Youth Top-notch Talent of Ten Thousand Talent Program (2014-253) and the National Natural Science Foundation of China (NSFC, 81170519).

## Conflict of Interest

The authors declare that the research was conducted in the absence of any commercial or financial relationships that could be construed as a potential conflict of interest.
