# Supplementary material for: Determining the Appropriate Treatment for T-Cell Acute Lymphoblastic Leukemia With SET-CAN/NUP214 Fusion: Perspectives From a Case Report and Literature Review
Source: Front Oncol. 2021 Mar 26;11:651494. doi: 10.3389/fonc.2021.651494 (PMC8044795; doi:10.3389/fonc.2021.651494)
Supplement: Supplementary file 1 [file Table_1.docx]

Supplementary table 1: RT-PCR assay covering 56 commonly detected fusion genes in leukemia

| *AML1-ETO* | *PML-RaRa(V)* | *FIP1L1-RARα* | *AML1-MDS1/EVI1* | *STRNE6-PDGFRA* | *KMT2A-AF1q* |
| --- | --- | --- | --- | --- | --- |
| *BCR-ABL（210）* | *NPM-RARα* | *TEL-AML1* | *AML1-MTG16* | *BCR-PDGFRA* | *KMT2A-AF4* |
| *BCR-ABL（190）* | *NUMA1-RARα* | *E2A-PBX1* | *ETV6-PDGFRA* | *TEL-PDGFRB* | *KMT2A-AF6* |
| *BCR-ABL（230）* | *PLZF-RARα* | *E2A-HLF* | *FIP1L1-PDGFRA* | *KMT2A-AF10* | *KMT2A-AF9* |
| *PML-RaRa（L）* | *PRKAR1A-RARα* | *SIL-TAL1* | *KIF5BE-PDGFRA* | *KMT2A-AF17* | *KMT2A-AFX* |
| *PML-RaRa(S)* | *STAT5b-RARα* | *DEK-CAN* | *CDK5RAP2-PDGFRA* | *KMT2A-AF1p* | *KMT2A-ELL* |
| *NUP98-HoxA13* | *NUP98-HoxA9* | *NUP98-HoxC11* | *NUP98-HoxD13* | *NUP98-PMX1* | *KMT2A-ENL* |
| *TEL-ABL* | *TEL-JAK2* | *TLS-ERG* | *NUP98-HoxA11* | *SET-CAN* | *KMT2A-SEPT6* |
| *WT1* | *HOX11* | *HOX11L2* | *CALM-AF10* | *HLXB9-ETV6* | *KMT2A-AF5* |
| *NPM-ALK* | *NPM-MLF1* |  |  |  |  |

Supplementary table 2: Next generation sequencing (NGS) assay of 118 commonly mutated genes in hematological disorders

| *ABL1* | *ASXL1* | *ATM* | *ATRX* | *BCL11B* | *BCL9* | *BCOR* | *BCORL1* | *BIRC3* | *BRAF* | *CALR* |
| --- | --- | --- | --- | --- | --- | --- | --- | --- | --- | --- |
| *CARD11* | *CBL* | *CBLB* | *CCND3* | *CD33* | *CDKN2A* | *CEBPA* | *CNOT3* | *CREBBP* | *CRLF2* | *CSF1R* |
| *CSF3R* | *CUX1* | *CXCR4* | *DDX41* | *DKC1* | *DLEU2* | *DNMT3A* | *DNMT3B* | *EGFR* | *ELANE* | *EP300* |
| *ERG* | *ETV6* | *EZH2* | *FANCA* | *FBXW7* | *FLT3* | *GATA1* | *GATA2* | *GNAS* | *GNB1* | *HLA-A* |
| *ID3* | *IDH1* | *IDH2* | *IKZF1* | *IL7R* | *IRF4* | *JAK1* | *JAK2* | *JAK3* | *KDM6A* | *KIT* |
| *KLF2* | *KRAS* | *MAP2K1* | *MAX* | *MEF2B* | *MLL* | *MPL* | *MYC* | *MYD88* | *NOTCH1* | *NOTCH2* |
| *NOTCH3* | *NPM1* | *NRAS* | *NT5C2* | *PAX5* | *PDGFRA* | *PHF6* | *PIK3CA* | *PIM1* | *PRPF40B* | *PTEN* |
| *PTPN11* | *RAD21* | *RBPJ* | *RET* | *RPL10* | *RPL11* | *RPL24* | *RPL35A* | *RPL5* | *RPS14* | *RPS19* |
| *RPS26* | *RUNX1* | *SETBP1* | *SETD2* | *SF1* | *SF3B1* | *SH2B3* | *SMC1A* | *SMC3* | *SRSF2* | *SRSF6* |
| *STAG2* | *STAT3* | *TERC* | *TERT* | *TET2* | *TINF2* | *TLE1* | *TLR2* | *TMEM14B* | *TNFAIP3* | *TP53* |
| *TRAF3* | *U2AF1* | *UTRN* | *WAS* | *WHSC1* | *WT1* | *XPO* | *ZRSR2* |  |  |  |

Supplementary table 3: In vitro drug sensitivity screening of refractory leukemia cells with SET/CAN fusion.

| **Drugs** | **Classification** | **Dosage** | **Inhibition rate (%)** |
| --- | --- | --- | --- |
| Carfilzomib | molecular targeted therapeutic drugs | 20 mg/m^2^ | 37.57 |
|  |  | 27 mg/m^2^ | 34.66 |
| Epirubicin | chemotherapy regimens | 120 mg/m^2^ | 32.97 |
|  |  | 60 mg/m^2^ | 27.11 |
| Mitoxantrone | chemotherapy regimens | 14 mg/m^2^ | 31.10 |
|  |  | 8 mg/m^2^ | 24.85 |
| cisplatin | chemotherapy regimens | 120 mg/m^2^ | 29.05 |
|  |  | 80 mg/m^2^ | 12.17 |
| Daunorubicin | chemotherapy regimens | 40 mg/m^2^ | 29.01 |
|  |  | 90 mg/m^2^ | 20.71 |
|  |  | 60 mg/m^2^ | 20.69 |
| Topotecan | chemotherapy regimens | 1.25 mg/m^2^ | 25.81 |
| Mycophenolate Mofetil) | immunoregulant | 0.5 g | 23.34 |
|  |  | 1.5 g | 15.36 |
| Venetoclax | molecular targeted therapeutic drugs | 400 mg | 22.86 |
|  |  | 100 mg | 16.37 |
|  |  | 20 mg | 8.88 |
| Pirarubicin | chemotherapy regimens | 20 mg/m^2^ | 22.61 |
|  |  | 40 mg/m^2^ | 19.13 |
| Teniposide | chemotherapy regimens | 60 mg/m^2^ | 22.57 |
|  |  | 30 mg/m^2^ | 1.05 |
| 5-Fluorouracil | chemotherapy regimens | 750 mg | 21.96 |
|  |  | 250 mg | 15.47 |
| Fludarabine | chemotherapy regimens | 25 mg/m^2^ | 21.64 |
| Bleomycin | chemotherapy regimens | 30 mg | 21.42 |
|  |  | 15 mg | 16.20 |
| Omacetaxine Mepesuccinate | chemotherapy regimens | 4 mg | 20.04 |
|  |  | 1 mg | 12.58 |
| Aclacinomycin | chemotherapy regimens | 1 mg/kg | 19.93 |
|  |  | 0.4 mg/kg | 9.64 |
| Bortezomib | molecular targeted therapeutic drugs | 1.3 mg/m^2^ | 19.51 |
| Mitomycin | chemotherapy regimens | 10 mg | 19.14 |
|  |  | 20 mg | 18.63 |
| Cyclophosphamide | chemotherapy regimens | 1000 mg/m^2^ | 18.90 |
|  |  | 500 mg/m^2^ | 3.64 |
| Doxorubicin | chemotherapy regimens | 60 mg/m^2^ | 18.09 |
|  |  | 40 mg/m^2^ | 13.70 |
| Etoposide | chemotherapy regimens | 60 mg/m^2^ | 18.06 |
|  |  | 100 mg/m^2^ | 15.73 |
| Cytarabine | chemotherapy regimens | 3000 mg/m^2^ | 17.25 |
|  |  | 2000 mg/m^2^ | 13.56 |
|  |  | 100 mg/m^2^ | 12.14 |
| Irinotecan | chemotherapy regimens | 350 mg/m^2^ | 16.29 |
| Dacarbazine | chemotherapy regimens | 200 mg/m^2^ | 15.58 |
|  |  | 400 mg/m^2^ | 7.01 |
| Lenalidomide | immunoregulant | 25 mg | 13.99 |
|  |  | 10 mg | 1.48 |
| Ixazomib | molecular targeted therapeutic drugs | 4 mg | 12.13 |
| Arsenic trioxide | chemotherapy regimens | 7 mg/m^2^ | 11.66 |
| Amsacrine | chemotherapy regimens | 120 mg/m^2^ | 11.24 |
| Docetaxel | chemotherapy regimens | 75 mg/m^2^ | 11.14 |
| Clofarabine | chemotherapy regimens | 50 mg/m^2^ | 11.06 |
| Carboplatin | chemotherapy regimens | 400 mg/m^2^ | 10.78 |
|  |  | 50 mg/m^2^ | <1 |
| Hydroxycamptothecin | chemotherapy regimens | 8 mg | 9.85 |
| Bexarotene | chemotherapy regimens | 300 mg/m^2^ | 8.65 |
|  |  | 400 mg/m^2^ | 8.25 |
| Cladribine | chemotherapy regimens | 0.09 mg/kg | 7.60 |
| Thalidomide | immunoregulant | 100 mg | 7.36 |
|  |  | 25 mg | 14.01 |
|  |  | 50 mg | 11.60 |
| Dasatinib | molecular targeted therapeutic drugs | 70 mg | 7.20 |
|  |  | 100 mg | 4.62 |
| Oxaliplatin | chemotherapy regimens | 130 mg/m^2^ | 6.75 |
| Paclitaxel | chemotherapy regimens | 200 mg/m^2^ | 5.75 |
|  |  | 135 mg/m^2^ | <1 |
| pomalidomide | immunoregulant | 4 mg | 5.24 |
| Desferrioxamine B | deferrization | 20 mg/kg | 4.10 |
| Vinorelbine | chemotherapy regimens | 30 mg/m^2^ | 3.89 |
| Chloroambucil | chemotherapy regimens | 0.1 mg/kg | 3.65 |
|  |  | 0.2 mg/kg | <1 |
| Isophosphamide | chemotherapy regimens | 1200 mg/m^2^ | 2.87 |
|  |  | 2500 mg/m^2^ | <1 |
| Sorafenib | molecular targeted therapeutic drugs | 400 mg | 2.47 |
| Vinblastine | chemotherapy regimens | 10 mg | 2.19 |
| Nilotinib | molecular targeted therapeutic drugs | 400 mg | 2.04 |
| Hydroxyurea | chemotherapy regimens | 20 mg/kg | 1.55 |
|  |  | 60 mg/kg | <1 |
| Adefovir Dipivoxil | chemotherapy regimens | 10 mg | 1.48 |
| Decitabine | demethylation | 15 mg/m^2^ | <1 |
|  |  | 20 mg/m^2^ | <1 |
| Vincristine | chemotherapy regimens | 1.4 mg/m^2^ | <1 |
| Ibrutinib | molecular targeted therapeutic drugs | 560 mg | <1 |
|  |  | 420 mg | <1 |
| Bosutinib | molecular targeted therapeutic drugs | 500 mg | <1 |
| Vindesine | chemotherapy regimens | 3 mg/m^2^ | <1 |
| Prednisone | glucocorticoid | 80 mg | <1 |
|  |  | 60 mg | <1 |
| Ruxolitinib | molecular targeted therapeutic drugs | 25 mg | <1 |
|  |  | 15 mg | <1 |
|  |  | 20 mg | <1 |
| Imatinib | molecular targeted therapeutic drugs | 400 mg | <1 |
|  |  | 600 mg | <1 |
| Deferasirox | deferrization | 20 mg/kg | <1 |
| Lomustine | chemotherapy regimens | 100 mg/m^2^ | <1 |
|  |  | 130 mg/m^2^ | <1 |
| Gemcitabine | chemotherapy regimens | 1000 mg/m^2^ | <1 |
|  |  | 1200 mg/m^2^ | <1 |
| Mercaptopurine | chemotherapy regimens | 80 mg/m^2^ | <1 |
|  |  | 100 mg/m^2^ | <1 |
| Methylprednisolone | glucocorticoid | 40 mg | <1 |
| Procarbazine | chemotherapy regimens | 50 mg | <1 |
| Tretinoin | chemotherapy regimens | 10 mg | <1 |
| Dexamethasone | glucocorticoid | 40 mg/m^2^ | <1 |
| Idarubicin | chemotherapy regimens | 7 mg/m^2^ | <1 |
|  |  | 8 mg/m^2^ | <1 |
| Bendamustine | chemotherapy regimens | 100 mg/m^2^ | <1 |
|  |  | 120 mg/m^2^ | <1 |
| Melphalan | chemotherapy regimens | 10 mg/m^2^ | <1 |
| Methotrexate | chemotherapy regimens | 10 mg | <1 |
| Busulfan | chemotherapy regimens | 0.8 mg/kg | <1 |
| Azacitidine | demethylation | 75 mg/m^2^ | <1 |
| Carmustine | chemotherapy regimens | 100 mg/m^2^ | <1 |
| Hydrocortisone | glucocorticoid | 100 mg | <1 |
| Midostaurin | molecular targeted therapeutic drugs | 50 mg | <1 |
|  |  | 100 mg | <1 |
| Chidamide | molecular targeted therapeutic drugs | 30 mg | <1 |
| Ponatinib | molecular targeted therapeutic drugs | 45 mg | <1 |
| Trametinib | molecular targeted therapeutic drugs | 2 mg | <1 |
| DAE | chemotherapy regimens | Daunorubicin 40 mg/m^2^  cytarabine 100 mg/m^2^   etoposide 100 mg/m^2^ | 45.19 |
| IAE | chemotherapy regimens | idarubicin 12 mg/m2  cytarabine 200 mg/m2   etoposide 100 mg/m2 | 40.02 |
| CHOEP | chemotherapy regimens | Cyclophosphamide 750 mg/m2  Epirubicin 70 mg/m2  Vincristine 1.4 mg/m2   etoposide 100 mg/m2  prednison; 60 mg/m2 | 31.21 |
| FA+VP | chemotherapy regimens | Fludarabine 30 mg/m2  cytarabine 2 g/m2  Vincristine 2 mg | 29.06 |
| MINE | chemotherapy regimens | Ifosfamide 1333 mg/m2  Mitoxantrone 8 mg/m2  etoposide 65 mg/m2 | 27.11 |
| ESHAP | chemotherapy regimens | etoposide 60 mg/m2  Methylprednisolone; 500 mg/m2  cytarabine 2 g/m2  cisplatin 25 mg/m2 | 27.05 |
| DOLP | chemotherapy regimens | Vincristine 2 mg  Daunorubicin 60 mg/m2  prednisone 60 mg  L-ASP 10000 u | 26.82 |
| Hyper-CVAD(B) | chemotherapy regimens | methotrexate 800 mg/m2  cytarabine 3000 mg/m2 | 25.67 |
| MA | chemotherapy regimens | Mitoxantrone 12 mg/m2  cytarabine 200 mg/m2 | 24.80 |
| DHAP | chemotherapy regimens | dexamethasone 40 mg  cytarabine 2000 mg/m2  cisplatin 100 mg/m2 | 24.08 |
| DA | chemotherapy regimens | Daunorubicin 60 mg/m2  cytarabine 100 mg/m2 | 23.72 |
| ME | chemotherapy regimens | Mitoxantrone 10 mg/m2  etoposide 100 mg/m2 | 22.64 |
| MOACD | chemotherapy regimens | Mitoxantrone 8 mg/m2  Vincristine 1.4 mg/m2  Cyclophosphamide 600 mg/m2  cytarabine 100 mg/m2  dexamethasone 6 mg/m2 | 21.02 |
| IOLP | chemotherapy regimens | Vincristine 1.4 mg/m2  idarubicin 10 mg  prednisone 60 mg/m2  L-ASP 10000 u | 18.43 |
| IA | chemotherapy regimens | idarubicin 12 mg/m2 cytarabine 100 mg/m2 | 17.29 |
| MOAP | chemotherapy regimens | cytarabine 1.0 g/m2  Mitoxantrone 10 mg/d  Vincristine 2 mg/d  prednisone 60 mg/d | 16.23 |
| HA | chemotherapy regimens | Homoharringtonine 2.5 mg/m2  cytarabine 100 mg/m2 | 15.65 |
| IOAP | chemotherapy regimens | Vincristine 1.4 mg/m2  idarubicin 10 mg/d  prednisone 60 mg/m2  cytarabine 100 mg/m2 | 14.11 |
| Mm | chemotherapy regimens | methotrexate 3 g/m2  6-mercaptopurine 25 mg/m2 | 13.88 |
| CLAG | chemotherapy regimens | Cladribine 5 mg/m2  cytarabine 2000 mg/m2 | 13.73 |
| DAC | chemotherapy regimens | Cladribine 5 mg/m2  cytarabine 200 mg/m2  Daunorubicin 60 mg/m2 | 12.86 |
| ICE | chemotherapy regimens | etoposide 100 mg/m2   Carboplatin 800 mg  Ifosfamide 5 g/m2 | 12.51 |
| TA | chemotherapy regimens | teniposide 100 mg/m2  cytarabine 100 mg/m2 | 12.45 |
| VTD | chemotherapy regimens | Bortezomib 1.3 mg/m2  Thalidomide 200 mg/d  dexamethasone 20 mg | 11.95 |
| DOMP | chemotherapy regimens | Vincristine 2 mg/d  Daunorubicin 60 mg  6-mercaptopurine 50 mg/d  prednisone 2 mg/kg/d | 11.25 |
| FLAG | chemotherapy regimens | Fludarabine 30 mg/m2  cytarabine 2 g/m2 | 10.21 |
| DAT | chemotherapy regimens | Daunorubicin 25 mg/m2  cytarabine 100 mg/m2  6-mercaptopurine 100 mg/m2 | 9.86 |
| HD-DA | chemotherapy regimens | cytarabine 3000 mg/m2  Daunorubicin 30 mg/m2 | 8.28 |
| VDLD | chemotherapy regimens | Vincristine 1.5 mg/m2  Daunorubicin 30 mg/m2  dexamethasone 8 mg/m2 L-ASP 5000 u/m2 | 8.22 |
| COATD | chemotherapy regimens | Cyclophosphamide 750 mg/m2 Vincristine 1.4 mg/m2  cytarabine 100 mg/m2 teniposide 100 mg/m2  dexamethasone 6 mg/m2 | 7.23 |
| VDCLP | chemotherapy regimens | Vincristine 1.4 mg/m2  Daunorubicin 40 mg/m2  Cyclophosphamide 750 mg/m2  L-asparaginas 6000 IU/m2  prednison; 1 mg/kg/d | 6.63 |
| HOAP | chemotherapy regimens | Homoharringtonine 4 mg/d  Vincristine 2 mg/d  cytarabine100 mg/d  prednisone40 mg/d | 6.54 |
| HAD | chemotherapy regimens | Homoharringtonine 2 mg/m2  cytarabine 100 mg/m2 Daunorubicin 40 mg/m2 | 5.09 |
| CAM | chemotherapy regimens | Cyclophosphamide 750 mg/m2  cytarabine 100 mg/m2  6-mercaptopurine 60 mg/m2 | 5.03 |
| VDLP | chemotherapy regimens | Vincristine 1.5 mg/m2  Daunorubicin 30 mg/m2  prednison; 40 mg/m2  L-ASP 5000 u/m2 | 4.96 |
| DCAG | chemotherapy regimens | Decitabine 15 mg/m2  cytarabine 10 mg/m2  aclacinomycin 8 mg/m2 | 3.54 |
| COP | chemotherapy regimens | Vincristine 1.4 mg/m2  Cyclophosphamide 750 mg/m2  prednison; 60 mg/m2 | 3.23 |
| GDP | chemotherapy regimens | gemcitabine 1 g/m2  dexamethasone 40 mg  cisplatin 75 mg/m2 | 2.57 |
| Large dose MTX+L-ASP | chemotherapy regimens | methotrexate 3 g/m2  L-asparaginas 6000 IU/m2 | 2.14 |
| DA-EPOCH | chemotherapy regimens | etoposide 50 mg/m2  Vincristine 0.4 mg/m2 Doxorubicin 10 mg/m2  Cyclophosphamide 750 mg/m2  prednison; 60 mg/m2 | <1 |
| HAA | chemotherapy regimens | Homoharringtonine 2 mg/m2  cytarabine 100 mg/m2  aclacinomycin 20 mg | <1 |
| Hyper-CVAD(A) | chemotherapy regimens | Cyclophosphamide 300 mg/m2  Doxorubicin 50 mg/m2 Vincristine 1.4 mg/m2  dexamethasone 40 mg/d | <1 |
| CHOP | chemotherapy regimens | Cyclophosphamide 750 mg/m2  Doxorubicin 50 mg/m2  Vincristine 1.4 mg/m2  prednison; 60 mg/m2 | <1 |
| ABVD | chemotherapy regimens | Doxorubicin 25 mg/m2  Bleomycin 10 mg/m2  Vincristine 6 mg/m2  dacarbazine 375 mg/m2 | <1 |
| CAG | chemotherapy regimens | cytarabine 10 mg/m2  aclacinomycin 20 mg | <1 |
| 6-MP+MTX | chemotherapy regimens | 6-mercaptopurine 60 mg/m2  Methotrexate 20 mg/m2 | <1 |

Supplementary table 4. Clinical characteristics of 64 T-ALL patients with SET-CAN fusion.

| Auther | Frequency in T-ALL AND country | Case No. | S/A | WBC | Immunophenotype | Karyotype | FISH | Gene mutation | Induction treatment | Efficiency(CR=1, others=0) | Allo-HSCT(Yes=1;No=0) | Follow up(Alive=0,Death=1) | Time  (months) |
| --- | --- | --- | --- | --- | --- | --- | --- | --- | --- | --- | --- | --- | --- |
| VanVlierberghe, P.  (10) | Netherlands 3.3%(3/92); in children | 1 | F/15.3 | 213 | NA | NA | del(9)(q34.11q34.13) | NOTCH1 | NA | NA | NA | 0 | 83 |
|  |  | 2 | F/10.6 | 142 | NA | NA | del(9)(q34.11q34.13) | NOTCH1 | NA | NA | NA | 0 | 83 |
|  |  | 3 | F/17.1 | 15 | NA | NA | del(9)(q34.11q34.13) ；NUP214-ABL1 amplification | NOTCH1 | NA | NA | NA | 0 | 37 |
| Gorello, P.  (11) | Italian 4.6%(7/152); in adluts | 4 | M/38 | 24 | pre-T | 46,XY[15] | del(9)(q34)/ABL1 | NOTCH1mut,  FBW7 WT | 0904 | 1 | 1 | 1 | 29 |
|  |  | 5 | M/19 | 3.28 | pre-T | 46,XY[15] | del(6)(q16)/GRIK2;del(9)(q34)/ABL1;del(12p)/ETV6 | NOTCH1mut,  FBW7 NA | 0904 | 1 | 1 | 0 | 23 |
|  |  | 6 | M/47 | NA | cortical | NA | del(9)(q34)/ABL1 | NOTCH1 WT,  FBW7 mut | refuse | NA | 0 | NA | NA |
|  |  | 7 | F/27 | NA | pre-T | NA | del(9)(p21)/CDKN2A-B;del(9)(q34)/ABL1;del(11)(p13)/LMO2;del(11)(q14)/CALM | NOTCH1mut,  FBW7 WT | 0496 | 0 | 0 | 1 | 12 |
|  |  | 8 | M/19 | NA | pro-T | Normal | del(9)(q34)/ABL1;del(11)(p13)/LMO2;del(11)(q14)/CALM;del(12)(p13)/ETV6 | NOTCH1 WT,  FBW7 WT | 0904 | 1 | 0 | 0 | 3 |
|  |  | 9 | M/18 | NA | pre-T | NA | del(9)(q34)/ABL1; del(5)(q35)/TLX3 | NOTCH1 WT,  FBW7 WT | AIEOP | 1 | NA | 1 | 24 |
|  |  | 10 | M/23 | NA | pre-T | 46,XY[12] | del(9)(q34)/ABL1 | NOTCH1 WT,  FBW7 WT | 0496 | 1 | 1 | 1 | 17 |
| Lee, S. G.(12) | Korea | 11 | M/28 | 3.733 | CD5,CD7, CD33, CD34 | 47,XY,del(1)(p13p22),del(6)(q13q21),del(9)(q12),del(11)(q13),-12,add(15)(p11.2),del(16)(q22),+19,+mar[3]/46, XY [17] | NA | NA | VDCLP+ methotrexate | 0 | Scheduled | 0 | NA |
| Li, W. J.  (13,14) | China 4.2%(2/48) in children | 12 | M/11 | 6.4 | HLA-DR; CD33;CD117; CD5;CD7;CD2;cyCD3 | NA | NA | NA | BCH-2003 Ⅰ | 1 | 0 | 1 | 3 |
|  |  | 13 | M/8 | 99.6 | CD5;CD7;cy CD3;CD34;CD10 | NA | NA | NA | BCH-2003 Ⅰ | 1 | 0 | 0 | 30 |
| Wang, Q. (15) | China  6.3% (3/48) | 14 | M/20 | 34.1 | NA | 46XY[10] | NA | PHF6,NOTCH1 | NA | NA | NA | NA | NA |
|  |  | 15 | M/23 | 2.65 | NA | 46XY[5] | NA | PHF6,NOTCH1 | NA | NA | NA | NA | NA |
|  |  | 16 | M/45 | 33.3 | NA | 46XY[20] | NA | PHF6,NOTCH1 | NA | NA | NA | NA | NA |
| Chae, H. (6) | Korea 10%(4/40) in adults | 17 | F/55 | 24.43 | CD33,CD34, CD13,CD7, cy-CD3 | NA | del(9)(q34)/ABL1 | NA | NA | NA | 0 | 0 | 31 |
|  |  | 18 | M/32 | 18.04 | CD33,CD34,CD13, CD7, CD5, cy-CD3 | 46,XY,del(13)(q12q14) | del(9)(q34)/ABL1 | NA | NA | NA | 0 | 1 | 42 |
|  |  | 19 | M/32 | 39.06 | CD33,CD34,HLADR, CD7, cy-CD3 | 46,XY,del(6)(q21q23),del(12)(p11.2) | del(9)(q34)/ABL1 | NA | NA | NA | 0 | 1 | 21 |
|  |  | 20 | F/20 | 5.07 | CD33,CD34,CD7,CD5, CD8, Cy-CD3 | 46,XX,+del(3)(q11.2),del(12)(p13),-13,add(17) (p.11.2) | del(9)(q34)/ABL1 | NA | NA | NA | 1 | 0 | 33 |
| Dai, H. P. (16] | China 10.3%(6/58) in adults | 21 | M/20 | 34.1 | CD7,cCD3,CD13,CD33, CD34 | 46XY[20] | del(9)(q34)/ABL1 | PHF6,NOTCH1 | NA | NA | NA | 1 | 9 |
|  |  | 22 | F/56 | 6.81 | CD7,cCD3,CD33, CD34 | 92-93,XXXX,+1,+3, +4,+4,-5,-6,7,+ 10,-18,+dmin*3-4[CP10] | del(9)(q34)/ABL1 | PHF6（-），NOTCH1 NA | NA | NA | NA | NA | NA |
|  |  | 23 | M/23 | 2.65 | CD7,cCD3,CD33,CD34 | 46XY[19] | del(9)(q34)/ABL1 | PHF6,NOTCH1 | NA | NA | NA | 0 | 17.8 |
|  |  | 24 | M/27 | NA | CD7,cCD3,CD13,CD33, CD34 | 46XY[20] | NA | NOTCH1 | NA | NA | NA | 1 | 15 |
|  |  | 25 | M/45 | 33.3 | CD7,cCD3, CD34 | 46XY[20] | NA | PHF6,NOTCH1 | NA | NA | NA | 1 | 30 |
|  |  | 26 | M/23 | 15.1 | CD7,cCD3,CD10,CD33, CD34 | 46XY[10] | del(9)(q34)/ABL1 | PHF6,NOTCH1 | NA | NA | NA | NA | NA |
| Lee, E. Y. (17) | Korea | 27 | F/43 | 60.6 | CD3, CD5, CD7, CD13, CD33, CD34 | 46,XX,dup(1)(p22p36.1) | del(9)(q34)/ABL1 | NA | NA | NA | NA | NA | NA |
| Liu, F.  (18) | China | 28 | NA | NA | NA | NA | **NA** | KRAS | NA | 0 | NA | NA | NA |
|  |  | 29 | NA | NA | NA | NA | NA | KRAS | NA | 0 | NA | NA | NA |
| Ben Abdelali, R. (9) | France 5.6%（11/196）; in adults | 30 | M/34 | 30.4 | cCD3,CD7,CD5,CD33,CD123,CD34 | 46, XY,t(3;10)(q?;q?) [20] | NA | NA | GRAALL 03（05） | 1 | 1 | 1 | 49 |
|  |  | 31 | F/37 | 8.6 | cCD3,CD7,CD5,CD123,CD34 | 46,XX,t(4;16)(q2?6;q23)[30] | NA | NA | GRAALL 03（05） | 1 | 1 | 0 | 64 |
|  |  | 32 | M/29 | 10.1 | cCD3,CD7,CD5,CD13,CD33,CD34 | 46,XY,del(6)(q14q24),del(11)(q21),del(12)(p12)[9]/46,XY[3] | NA | NA | GRAALL 03（05） | 1 | 1 | 0 | 44 |
|  |  | 33 | M/41 | 18.4 | cCD3,CD7, CD33,CD34, CD117 | 47,XY,+4[15] | NA | NA | GRAALL 03（05） | 1 | 1 | 0 | 46 |
|  |  | 34 | M/23 | 604.4 | cCD3,CD7,CD2,D5,CD4,CD8 | 46,XY[31] | NA | NA | GRAALL 03（05） | 0 | 0 | 1 | 5 |
|  |  | 35 | M/30 | 24.9 | cCD3,sCD3,CD7,CD5 | 46,XY[21] | NA | NA | GRAALL 03（05） | 1 | 1 | 0 | 66 |
|  |  | 36 | M/36 | 181.8 | cCD3,CD7,CD5,CD33,CD34,HLA-DR | 46,XY,add(5)(q22),del(12)(p11p13)[2]/46,XY,der(5)t(5;12)(q11.2;p13),del(12)(p11p13),der(12)t(5;12)(q11.2;p13)add(5)(q22)[2] /46,XY[16] | NA | NA | GRAALL 03（05） | 1 | 1 | 0 | 24 |
|  |  | 37 | M/45 | 50.8 | cCD3,CD7,CD2,D5,CD4,CD8 | 46,XY,del(5)(q?q?)[7]/46,XY,del(13)(q12q14),inv(14)(q11q32),del(16)(p12p13.3)[5]/46,XY[5] | NA | NA | GRAALL 03（05） | 1 | 0 | 0 | 33 |
|  |  | 38 | M/38 | 2.8 | cCD3,CD7,CD5, CD33,CD123,CD34,CD117 | 88,XX,-Y,-Y,[4n],add(2)(q24),+4,-5,-5,add(5)(q?35),-7,-9,add(9)(p21),del(9)(q11q12),+10,del(12)(p13)x2,-17x2,+2mar[cp7]/ 77~89,sl,+Y,+Y,-add(9),-del(9),+9,+9,+1~2mar[cp3]/78~88,sdl1,-9,add(15)(p11)[cp6]/46,XY[1] | NA | NA | GRAALL 03（05） | 1 | 1 | 1 | 9 |
|  |  | 39 | M/28 | 41.8 | cCD3,CD7,CD2,CD5, CD33 ,CD34,CD117,HLA-DR | 46,XY,del(5)(q31q35),del(6)(q?12q?16),del(7)(q34),del(12)(p12),del(16)(q2?)[29]/47,idem,del(11q),+mar[6]/46,XY[3] | NA | NA | GRAALL 03（05） | 1 | 1 | 0 | 30 |
|  |  | 40 | M/20 | 30.9 | cCD3,CD7,CD5 | 48,XY,+21,+21 [5] / 46,XY [25] | NA | NA | GRAALL 03（05） | 1 | 1 | 0 | 28 |
| Prokopiou, C. (19) | Cyprus 4.9%(2/41)  In ALL adults | 41 | F/48 | NA | CD7+,CD5dim,cCD3+, CD34+,HLADR+, CD117+, MPO+ | complex karyotype(-12,-13,-17,-18) | NA | NA | NA | NA | 1 | 1 | 12 |
|  |  | 42 | M/45 | NA | CD7+, CD38+, CD34+, CD3+, CD33+ | NA | NA | NA | NA | NA | 1 | 1 | 6 |
| Yunhong Wang （20） | China 14.3%(1/7) in adults | 43 | M/37 | NA | NA | 46,XY,del(7)(q22q35)[17]/46,XY[3] | Normal | NA | NA | NA | NA | NA | NA |
| Dong Xiaoyan （21） | China  3.57% (1/28) | 44 | M/14 | 135.4 | CD34,CD38,CD10,CD5,CD33,CD7,cCD3,CD71,CD99,TdT | 46,XY,add(2)(q37),del(11)(q21)[10]/46XY[10] | NA | NA | MEA | 0 | 0 | 0 | 2 |
| Chen, B. （8） | China 13.1% in adults (8/61)  2.9% in children(2/69) | 45 | M/24 | 0.29 | pro-T | 46,XY | NA | NOTCH1,PHF6,EVL, STAT5B, JAK3, DNM2 | VDPCP | 1 | NA | NA | NA |
|  |  | 46 | M/52 | 8.8 | ETP-ALL | 46,XY,-7,+dm[1]/[4] | NA | IDH1,NOTCH1,IDH2,TDP1,EED,IKZF1,BCOR,SUZ12 | VDPCP | 0 | NA | NA | NA |
|  |  | 47 | F/46 | 13.6 | cortical-T-ALL | NA | NA | NOTCH2,STAT5B,SUZ12,KRAS,CHD4,MINK1 | VDPCP | 0 | NA | NA | NA |
|  |  | 48 | M/45 | 1.7 | ETP-ALL | 46,XY | NA | NOTCH1,NUMA1,KMT2D,ZNF573,KMT2B,ARID1A,KRAS,CHD4 | VDPCP | 1 | NA | NA | NA |
|  |  | 49 | M/28 | 60.7 | ETP-ALL | 47,XY,+21[3]/46,XY[8]/[19] | NA | PTPRC,EZH2,NOTCH1,PHF6,JAK1,CCND3,JAK3 | VDPCP | 1 | NA | NA | NA |
|  |  | 50 | M/26 | 1.17 | ETP-ALL | 46,XY,t(1;2)[1]/41~46,XY,t(1;2),7p-[CP2]/39~46,XY,7p-[3]/46,XY[6]/[20] | NA | FBXW7,NOTCH1,NRAS,CENPE,CEBPA | VDPCP | 1 | NA | NA | NA |
|  |  | 51 | F/41 | 15.8 | ETP-ALL | 46,XX | NA | MAD2L1,EED,JAK1,KMT2D,CCND3,KRAS,JAK3,BOD1L1 | VDPCP | 1 | NA | NA | NA |
|  |  | 52 | M/29 | 42 | ETP-ALL | 46,XY,t(15;17)(q26;q11)[2]/46,XY[2] | NA | NOTCH1,PHF6,ASXL1 | VDPCP | 1 | NA | NA | NA |
|  |  | 53 | M/11 | 1.1 | ETP-ALL | NA | NA | EZH2,NOTCH1,PHF6,PTEN | SCMC-ALL-2005 | 1 | NA | NA | NA |
|  |  | 54 | M/12 | 129.5 | ETP-ALL | 46,XY | NA | FBXW7,CCND3,ZNF573,KRAS,MINK1 | SCMC-ALL-2005 | 1 | NA | NA | NA |
| Papenhausen, P. （22） | America 15%（6/40）in children | 55 | M/14 | NA | NA | 45,X,-Y,?add(1)(p36.2), del(1)(q35),−5, del(11) (q23.1q23.3), +17, der(17)t(5;17)(p12;p11.2)x2 [CP16]/[46,XY[4] | del(9)(q34)/ASS1,ABL1;del(12)(p13)/ETV6;+17/D17Z1 | NA | NA | NA | NA | NA | NA |
|  |  | 56 | NA/8 | NA | NA | NA | del(9)(q34)/ASS1,ABL1; | NA | NA | NA | NA | NA | NA |
|  |  | 57 | F/13 | NA | NA | 46,XX | del(9)(q34)/ASS1,ABL1; | NA | NA | NA | NA | NA | NA |
|  |  | 58 | M/10 | NA | NA | 46,XY | del(9)(q34)/ASS1,ABL1; | NA | NA | NA | NA | NA | NA |
|  |  | 59 | F/4 | NA | NA | 46,XX,add(1)(p12),-5, del(6)(q13q16),add(7)(q32),del(9) (q22q34), add(12)(p11.2),+mar[cp6]/ 46,XX[5] | del(9)(q34)/ASS1,ABL1;del(12)(p13)/ETV6; | NA | NA | NA | NA | NA | NA |
|  |  | 60 | M/17 | NA | NA | 47,XY, + 4,del(12) (p11.2),add(12)(q24.1)[12]/ 46,XY[9] | del(9)(q34)/ASS1,ABL1;del(12)(p13)/ETV6;+4/D4Z1 | NA | NA | NA | NA | NA | NA |
| Kwock, J. M. （23） | America | 61 | M/14 | NA | cCD3+,CD2+,CD5+,CD7+,TdT+, and CD10+ | NA | del(9)(q34)/ABL1; | NA | NA | NA | NA | NA | NA |
| Yang, Q. （7） | China | 62 | M/26 | 12.3 | CD7,CD99, cCD3,CD33, CD34, CD10,CD2 | 46, XY, del(11)(q13), del(13)(q14),inv(16)(p13.3q23) | NA | NA | VICP | NA | 0 | 1 | 0.5 |
|  |  | 63 | M/51 | 109.1 | CD7,CD33,CD99,CD10,CD34,cCD3,CD5, cTDT | NA | NA | NA | VICP(14d)+MEA | NA | 0 | 1 | 1.2 |
|  |  | 64 | M/37 | 131.5 | CD7,CD99,CD38,CD34,CD33,HLA-DR, cCD3,cTDT | 45, XY, der(17;19)(q10; q10)/46,XY) | NA | NA | CALGB9111(14d)+CLAG+L | 0 | 0 | 0 | 10 |
| Zhang, H. （1） | China | 65 | M/21 | 37.16 | CD7, cCD3, CD38, CD5, HLA-DR | 46,XY[20] | del(9)(q34)/ABL1; | BRAF | VICP | 1 | 1 | 0 | 12 |
| Na Lin  (this case) | China | 66 | F/15 | 23.5 | CD7,CD38,CD2,CD11,cCD3,CD99,CD5,CD34,CD10 | 46,XX,del(12)(p11)[19]/46,XX[1] | NA | NOTCH1, JAK1, JAK3, NRAS, DNM2 | VICLP | 0 | 1 | 0 | 14 |
| Chen, X. （25） | China  2.92%(4/137) in adults  0.39%(1/256) in children | 67-71 | NA | NA | NA | NA | NA | NA | NA | NA | NA | NA | NA |
